# Pancreatic arteriovenous malformation: A multi-modal diagnostic approach

**DOI:** 10.1016/j.radcr.2026.03.059

**Published:** 2026-04-24

**Authors:** Samyak Shah, James Liu, Peter Tagkalidis, Marty Smith, Charles H.C. Pilgrim, Bruno Di Muzio

**Affiliations:** a Department of Radiology, Alfred Hospital, 55 Commercial Road, Melbourne, VIC 3004, Australia; b Department of Hepatopancreatobiliary Surgery, Alfred Hospital, 55 Commercial Road, Melbourne, VIC 3004, Australia; c Department of Gastroenterology, Alfred Hospital, 55 Commercial Road, Melbourne, VIC 3004, Australia; d School of Translational Medicine, Monash University, Wellington Road, Clayton, VIC 3800, Australia; e School of Public Health and Preventative Medicine, Monash University, 553 St Kilda Road, VIC 3004, Australia

**Keywords:** Pancreatic arteriovenous malformation, Computed tomography, Magnetic resonance imaging, 68Ga-DOTATATE positron emission tomography, Endoscopic ultrasound, Pancreatic neuroendocrine tumour

## Abstract

Pancreatic arteriovenous malformation is exceptionally rare with fewer than 200 cases reported in the literature. It is usually asymptomatic, although patients can present with abdominal pain, gastrointestinal bleeding, or portal hypertension. Their radiological features can closely mimic those of other hypervascular pancreatic lesions, particularly pancreatic neuroendocrine tumours, making accurate diagnosis challenging. We report the case of a 77-year-old patient with incidentally detected hypervascular pancreatic nodules and the importance of a multimodal approach in establishing the diagnosis of pancreatic arteriovenous malformation.

## Introduction

Pancreatic arteriovenous malformation (pAVM) is an extremely rare vascular anomaly, most often congenital, representing only about 0.9% of gastrointestinal vascular malformations [1,2]. The majority of reported cases in the literature describe pAVMs as solitary lesions without associated vascular anomalies such as aneurysms or other malformations. Whilst most patients remain asymptomatic, some may develop abdominal pain; in more severe cases, pAVM can manifest with gastrointestinal bleeding, acute pancreatitis, or refractory portal hypertension, underscoring the importance of accurate diagnosis [3,4].

Although advancements in cross-sectional imaging have improved diagnostic precision, pAVMs can still mimic other pancreatic lesions, making differentiation sometimes challenging [5]. We present a case that illustrates the value of a multimodal approach, including computed tomography (CT), magnetic resonance imaging (MRI), Gallium-68 (68Ga) DOTATATE PET/CT scan, and endoscopic ultrasound (EUS), in establishing the diagnosis of pAVM whilst excluding alternative pathologies.

## Case report

**Fig. 1:**
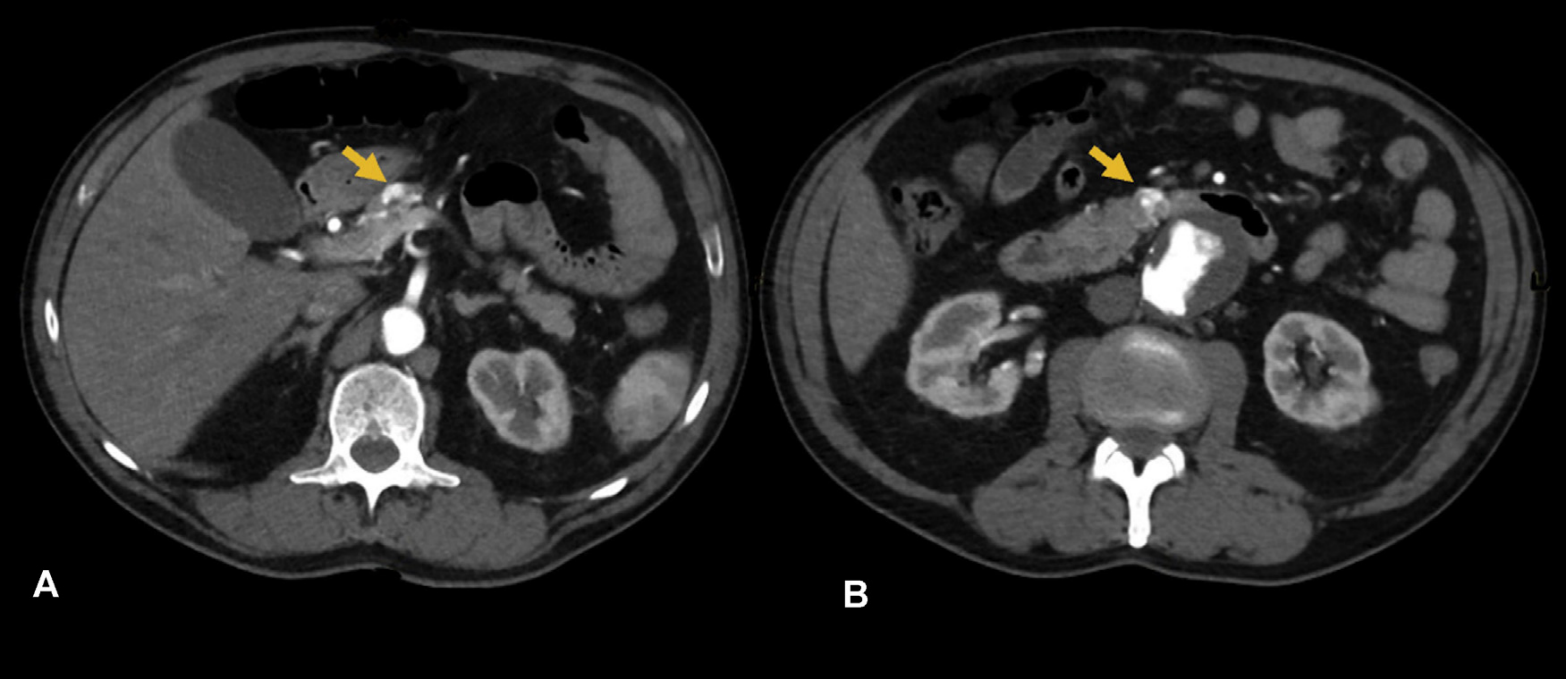
Axial arterial phase CT aortogram demonstrating 2 subcentimetre hypervascular foci (arrows) within the pancreatic parenchyma, relatively circumscribed but with irregular margins: (A) superior aspect of the pancreatic neck; (B) anteromedially within the uncinate process.

A 77-year-old male was referred to the Hepatopancreatobiliary (HPB) Surgery Unit for further investigation of small hypervascular pancreatic lesions that were incidentally detected during an admission for rigors and back pain. A CT aortogram (CTA), which was performed to exclude an acute aortic pathology, demonstrated 2 hypervascular nodules measuring 11 mm and 10mm at the pancreatic neck and uncinate process respectively (Fig. 1A and B). His past medical history was significant for hypertension, a recent diagnosis of a 46mm infrarenal fusiform aortic aneurysm, and a 50 pack-year smoking history.

**Fig. 2:**
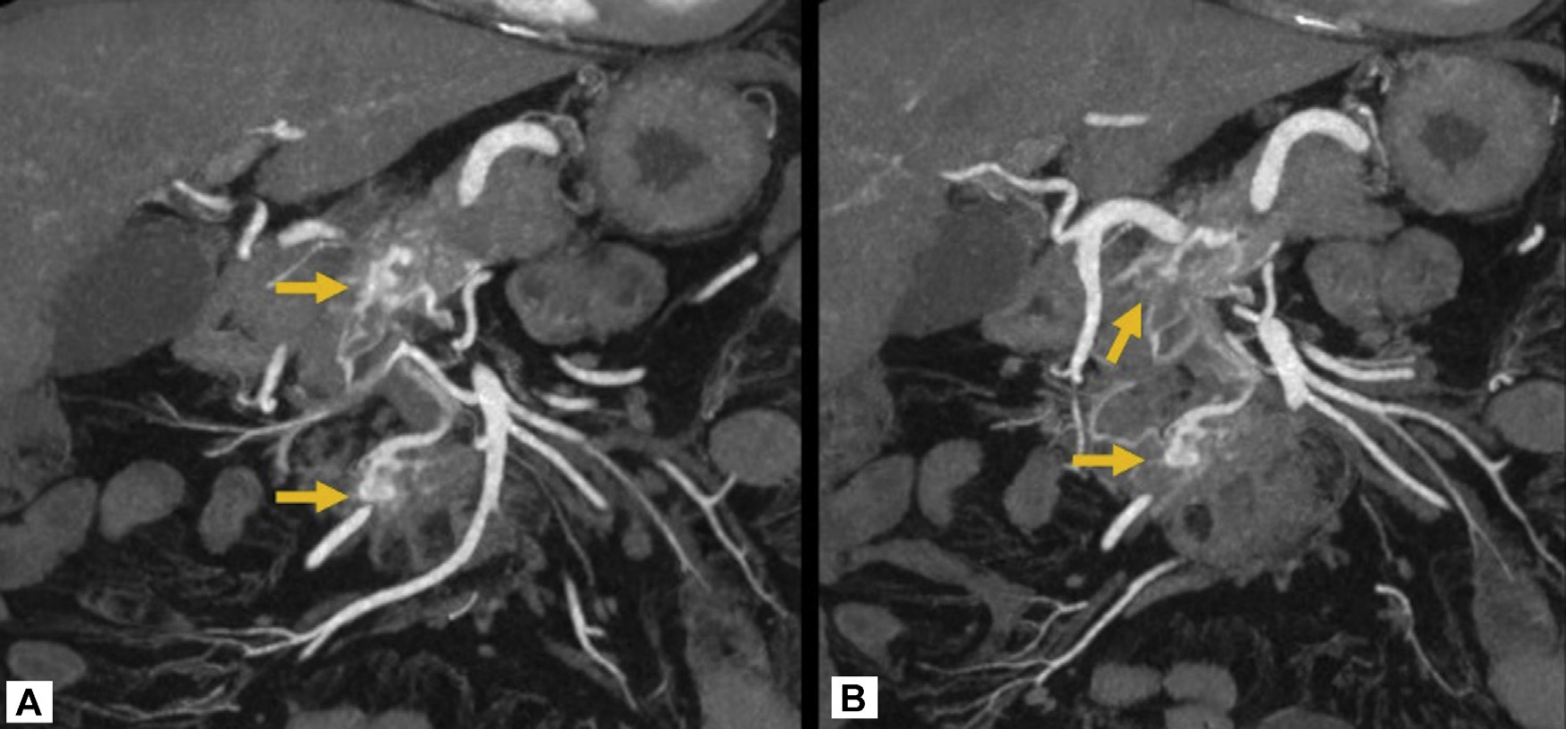
Coronal maximum intensity projection (MIP) reformats of arterial phase CT aortogram better demonstrate the irregular morphology of the hypervascular foci (arrows) within the pancreatic parenchyma, which appear serpentine and nidus-like, with communication to small arterial branches, suggestive of an arteriovenous malformation. The focus at the pancreatic neck is supplied by the anterior superior pancreaticoduodenal artery, whereas the lesion in the uncinate process appears to arise from a branch of the anterior inferior pancreaticoduodenal artery. (A) and (B) represent sequential slices from anterior to posterior.

**Fig. 3:**
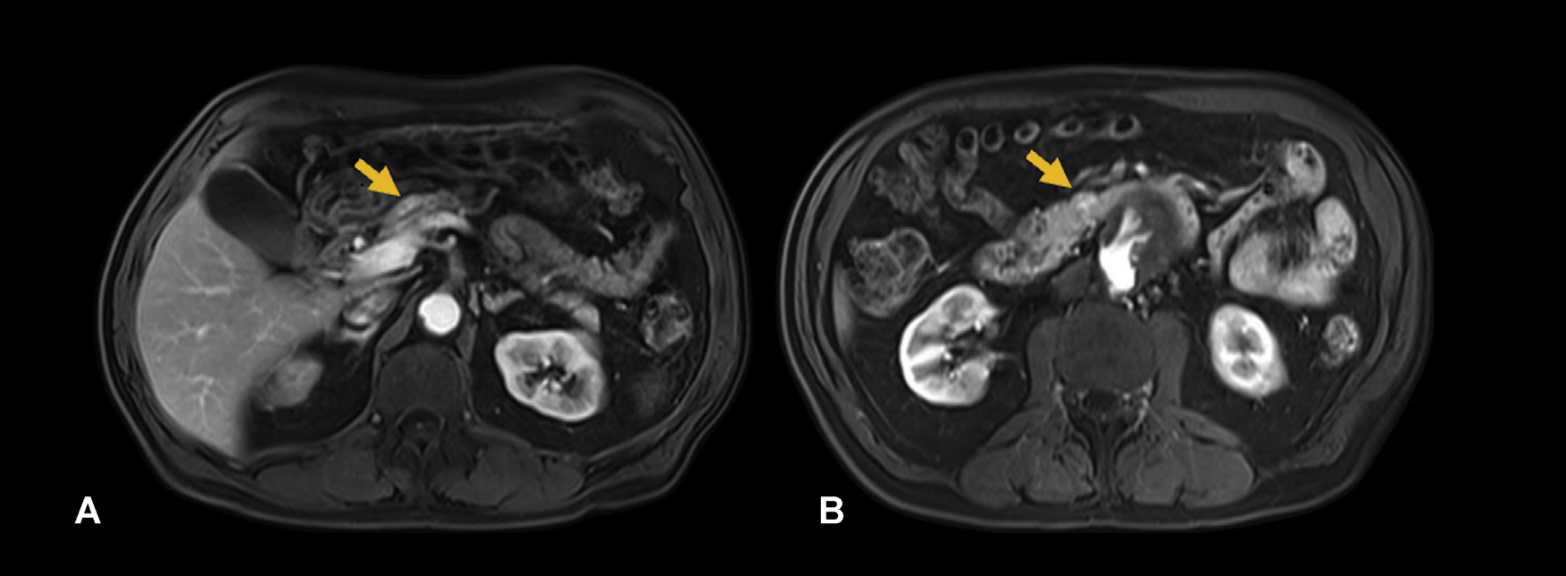
Axial arterial-phase T1-weighted fat-saturated MRI demonstrates the pancreatic neck (A) and uncinate process (B) lesions (arrows) at the same anatomical level as the CT images for comparison. The lesions are less conspicuous than on CTA and show no correlate on T2-weighted imaging or DWI/ADC (not shown).

Upon follow-up in the HPB Clinic 4 weeks after his admission, he was asymptomatic, with no abdominal pain, weight loss, or other gastrointestinal symptoms. On examination, he was not jaundiced, and his abdomen was non-tender with no palpable masses. A pancreatic MRI was subsequently performed, given the suspicion for incidental pancreatic neuroendocrine tumours (pNET) or, less likely, hypervascular metastases from an unknown primary. This again demonstrated both the arterial hypervascular lesions at the pancreatic neck (Fig. 2A) and uncinate process (Fig. 2B). These lesions were less conspicuous on MRI arterial-phase T1-weighted imaging (∼25-35s) due to strong background pancreatic parenchymal enhancement (Fig. 3), whereas they were more clearly visualised on CTA arterial-phase imaging (∼15-25s). They were not visualised on other MRI sequences, including T2-weighted, fat-suppressed T1-weighted, and diffusion-weighted imaging. MRI was performed on a 3T system using intravenous gadolinium-based contrast (Gadovist); sequences included coronal 2D FIESTA Fat Sat, axial 2D FIESTA Fat Sat, axial T1 In/Out Phase, axial DWI, axial T2, axial T2 Fat Sat, and multiphasic axial T1 LAVA C+ (arterial, portal, venous, and 5-minute delayed phases). There were no signs of primary malignancy in other viscera, particularly the kidneys.

Laboratory evaluation included a neuroendocrine tumour screen. Liver function tests were largely unremarkable apart from a mildly elevated ALT. Biochemical markers demonstrated elevations in pancreatic polypeptide (379.2pmol/L; normal <100), chromogranin A (207mcg/L; normal <101), vasoactive intestinal peptide (VIP) (74.2pmol/L; normal 7.3–25.4), and glucagon (229pg/mL; normal ≤208), while other markers including gastrin, serotonin, insulin, and urine 5-HIAA were within normal limits. The patient had impaired renal function (eGFR ∼35). Overall, these abnormalities were mild, nonspecific, and did not convincingly support the presence of a functional pNET.

**Fig. 4:**
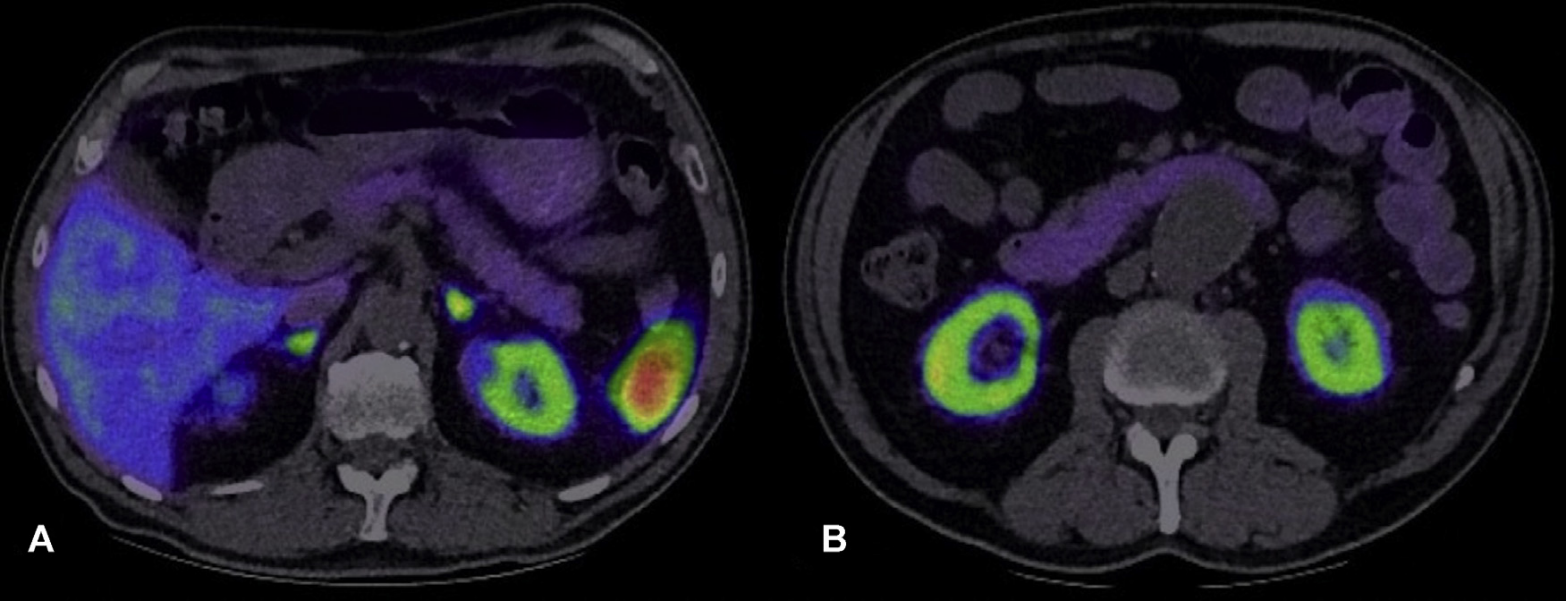
Axial 68Ga-DOTATATE PET/CT performed to investigate suspected pNET demonstrates no abnormal tracer uptake within the pancreas. (A) and (B) correspond to the axial levels where the lesions had been identified on CT.

Both the CT and MRI were interpreted as concerning for multifocal pNET. In the context of these radiological findings and marginal elevations in selected neuroendocrine tumour markers, further functional characterisation was warranted. Accordingly, a 68Ga-DOTATATE PET/CT was performed 3 weeks after the MRI. This study demonstrated no abnormal tracer uptake in the pancreas (Fig. 4).

**Fig. 5:**
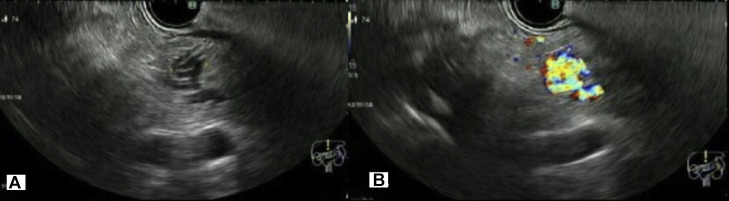
Endoscopic ultrasound identified only the lesion at the pancreatic neck. (A) B-mode image of the pancreatic neck demonstrates a cluster of tortuous, tubular, anechoic structures. (B) Color Doppler shows turbulent vascular flow consistent with arteriovenous malformation.

Subsequent discussion at the multidisciplinary team meeting, including subspecialty abdominal radiologists, highlighted the atypical morphology of the pancreatic lesions on CT and MRI. Multiplanar reconstructions on CT, particularly on the coronal plane (Fig. 2), suggested a cluster of vessels with serpentine configuration in the location of the lesions, more in keeping with arteriovenous malformations (AVMs). Gastrointestinal EUS was ultimately recommended and performed, identifying an 11 mm vascular malformation in the pancreatic neck anterior to the main pancreatic duct, corresponding to the arterially enhancing lesion (Fig. 5A). This comprised small tortuous vessels with an identifiable feeding vessel, and colour Doppler confirmed turbulent vascular flow consistent with AVM (Fig. 5B). No discrete mass was demonstrated, and no corresponding lesion was identified in the uncinate process, most likely due to anatomical and technical limitations of this region. Standard grayscale EUS may not reliably detect small hypervascular lesions, whereas contrast-enhanced EUS or multiphase CT/MRI can improve detection. Given the absence of symptoms, no intervention was required. His AVMs will continue to be monitored with surveillance imaging alongside his asymptomatic infrarenal aortic aneurysm. Surveillance will be performed with CT aortogram as per the vascular unit’s recommendation, with the next scan planned in one year.

## Discussion

pAVMs are highly uncommon, with fewer than 200 cases reported in the literature since the first description by Halpern et al in 1968[6]. They can be classified into congenital and acquired. Approximately 90% are congenital in origin, arising from abnormal development of the arteriovenous plexus during embryogenesis. Of these, 10-30% are thought to be associated with Rendu-Osler-Weber syndrome, also known as hereditary haemorrhagic telangiectasia, an autosomal dominant inherited disorder [7]. Acquired pAVMs may develop secondary to trauma, pancreatitis, tumours, medical interventions, or other factors [7,8]. pAVMs are most commonly located in the pancreatic head (59.4%), as in our case, followed by the body and tail (33.3%); while involvement of the entire pancreas occurs in 7.2% of cases [9,12]. These malformations feature abnormal arteriovenous shunts within the pancreas, resulting in atypical vascular proliferation and altered blood flow between the arterial and portal venous systems [3].

Intervention is primarily indicated in symptomatic cases, including those presenting with abdominal pain, gastrointestinal bleeding, pancreatitis, or portal hypertension. Other indications include evidence of lesion growth or high-risk features that suggest an increased risk of catastrophic bleeding or other complications. Surgical resection is generally preferred in symptomatic patients, as it provides definitive management and reduces the risk of recurrence or further complications [9]. Transarterial embolisation may be considered in patients at high surgical risk, although it can be challenging when pAVMs are supplied by numerous feeding arteries and may result in incomplete treatment [10,11].

Asymptomatic patients with incidentally detected pAVMs, as in this case, are most often managed conservatively, particularly when the lesion is stable and lacks high-risk features. Given the rarity of this condition, management is largely guided by expert opinion and institutional experience rather than formal consensus guidelines. Multidisciplinary evaluation is recommended, with referral to pancreatic specialists for complex or high-risk cases. Surveillance using Doppler ultrasound, CT Angiography or MRI can be considered for lesions with uncertain risk or to monitor for progression, with intervals typically tailored to individual lesion characteristics. pAVMs, particularly when small and nidus-like, can closely mimic other hypervascular pancreatic lesions, making accurate diagnosis challenging. Pancreatic neuroendocrine tumours (pNETs) represent the most important differential, as they typically appear as well-circumscribed round or oval hypervascular lesions and may be multiple or incidentally detected in asymptomatic patients [15]. In contrast, pAVMs may demonstrate a more irregular or tubular morphology with serpiginous vascular channels and connections to adjacent vessels, reflecting abnormal arteriovenous shunting. However, intense arterial enhancement may produce contrast “blooming” on standard window settings, potentially obscuring the underlying serpiginous vascular configuration and causing the lesion to resemble a solid hypervascular mass. Other considerations include splenic artery aneurysm or pseudoaneurysm, intrapancreatic accessory spleen which typically manifests as a hypervascular nodule in the distal pancreas, as well as hypervascular metastases, most often from renal cell carcinoma [18].

As pAVMs are rare and can resemble other hypervascular pancreatic lesions, diagnosis often requires a multimodal imaging approach. CT is considered the primary imaging modality for evaluation. Typical findings include a nidus-like cluster of hyperenhancing vascular structures within the pancreas, which may appear as punctate foci or even a mass-like focus on thicker slices or due to contrast blooming on standard window settings. Careful interrogation with thin-slice reconstructions and post-processing can resolve these into tubular or serpiginous intrapancreatic vessels representing the vascular nidus. CT is also highly sensitive for identifying early portal venous filling during the arterial phase (Fig. 1A), a hallmark of arteriovenous shunting [1,12]. Thin-slice acquisition (≤1 mm) with submillimetre collimation and advanced post-processing techniques, including multiplanar reformats (MPR) and maximum intensity projections (MIP), improve visualisation of serpentine vascular channels, facilitate recognition of early draining veins, and enable robust 3D vascular mapping. These tools enhance diagnostic confidence and are particularly valuable in distinguishing pAVM from other hypervascular pancreatic lesions and could be applied retrospectively in our case.

MRI typically depicts multiple signal voids in pAVMs due to high-velocity flow through dilated vessels, seen as serpentine or tubular areas of absent signal on both T1- and T2-weighted sequences. These flow voids reflect rapid intravascular flow that prevents generation of MR signal within the involved vessels, and they are considered a hallmark feature of high-flow vascular malformations such as AVMs [8,13]. In our case, however, this characteristic finding was not demonstrated, likely owing to the small size of the lesions, the relatively large field of view used in the MRI protocol (which limited spatial resolution), and artefacts related to the proximity of the pancreas to the stomach and adjacent bowel.

Furthermore, as demonstrated in our case, functional imaging with 68Ga-DOTATATE PET/CT can play a key role in refining the diagnosis. This modality has been reported to demonstrate a sensitivity exceeding 95% for well-differentiated pNETs, making it a highly reliable tool when clinical or radiological suspicion exists[14]. Nevertheless, detection of very small lesions may still be limited by the intrinsic spatial resolution of PET imaging (approximately 3–6 mm), and subcentimeter lesions may therefore occasionally be missed despite otherwise high diagnostic performance. Its use can, however, substantially increase diagnostic confidence when distinguishing pNETs from other hypervascular pancreatic lesions.

Endoscopic ultrasound (EUS) is frequently used in the evaluation of indeterminate pancreatic lesions due to its high spatial resolution and the ability to obtain tissue sampling when required. Although not routinely performed for suspected pancreatic arteriovenous malformations, the patient in our case underwent EUS, which contributed to establishing the diagnosis. The findings were consistent with those described by Hu et al., demonstrating a circumscribed anechoic lesion in the pancreatic head with colour Doppler revealing tortuous, dilated vascular channels and a characteristic mosaic or turbulent flow pattern [16].

Digital subtraction angiography (DSA), although not performed in our case, can provide detailed anatomical and hemodynamic information and allows direct visualization of the nidus, feeding arteries, and early draining veins. It is generally reserved for situations where non-invasive imaging is equivocal, or for pre-interventional planning [8,17]. However, with modern high-resolution multiphase CT angiography, many of these features can now be reliably demonstrated noninvasively, reducing the role of diagnostic angiography and reserving DSA primarily for therapeutic intervention.

Mild elevations in selected neuroendocrine tumour markers further contributed to the diagnostic uncertainty in this case. However, such biochemical abnormalities may occur in the absence of a functional pNET. Non-neoplastic causes include reduced renal clearance, chronic gastric mucosal disease, inflammatory states, and certain cardiovascular conditions [19]. In our patient, impaired renal function (eGFR ∼35) may have contributed to these nonspecific elevations, and the results were therefore interpreted cautiously in the clinical and imaging context. This case highlights the diagnostic challenges posed by pancreatic arteriovenous malformation, a rare entity that can closely mimic more common hypervascular pancreatic lesions such as pNET. In our patient, the initial CT and MRI appearances were interpreted as concerning for pNET, and borderline elevations in several neuroendocrine tumour markers further contributed to diagnostic uncertainty. However, the absence of uptake on 68Ga-DOTATATE PET/CT, together with multimodality imaging and multidisciplinary review, ultimately redirected suspicion towards a vascular malformation. Confirmation with EUS underscored the importance of a systematic, stepwise approach, while also illustrating how the rarity of this condition and overlapping features with pNET can confound diagnosis.

## Conclusion

pAVMs are rare vascular anomalies that pose significant diagnostic challenges, often mimicking other hypervascular pancreatic lesions such as pNETs. This case highlights the value of a multimodal imaging approach in achieving accurate recognition of pAVMs, thereby guiding appropriate management and avoiding unnecessary invasive procedures.

## Patient consent

Written, informed consent was obtained from the patient for their anonymised medical information to be included within the manuscript for publication.
